# Reflections on ‘medical tourism’ from the 2016 Global Healthcare Policy and Management Forum

**DOI:** 10.1186/s12919-017-0075-8

**Published:** 2017-07-13

**Authors:** Valorie A. Crooks, Meghann Ormond, Ki Nam Jin

**Affiliations:** 1 Department of Geography RCB 7227, Simon Fraser University, 8888 University Drive, Burnaby, BC V5A 1S6 Canada; 2 Cultural Geography Wageningen University & Research, PO Box 47, Wageningen, 6700 AA The Netherlands; 3 Department of Health Administration, Yonsei University, Wonju, South Korea

**Keywords:** Medical tourism, Medical travel, Research

## Abstract

In October 2016, the Global Healthcare Policy and Management Forum was held at Yonsei University, Seoul, South Korea. The goal of the forum was to discuss the role of the state in regulating and supporting the development of medical tourism. Forum attendees came from 10 countries. In this short report article, we identify key lessons from the forum that can inform the direction of future scholarly engagement with medical tourism. In so doing, we reference on-going scholarly debates about this global health services practice that have appeared in multiple venues, including this very journal. Key questions for future research emerging from the forum include: who should be meaningfully involved in identifying and defining categories of those travelling across borders for health services and what risks exist if certain voices are underrepresented in such a process; who does and does not ‘count’ as a medical tourist and what are the implications of such quantitative assessments; why have researchers not been able to address pressing knowledge gaps regarding the health equity impacts of medical tourism; and how do national-level polices and initiatives shape the ways in which medical tourism is unfolding in specific local centres and clinics? This short report as an important time capsule that summarises the current state of medical tourism research knowledge as articulated by the thought leaders in attendance at the forum while also pushing for research growth.

## Introduction

On 18–19 October 2016, the Global Healthcare Policy and Management Forum (GHPMF) was held at Yonsei University, Seoul, South Korea. The goal of the forum was to discuss the role of the state in regulating and supporting the development of medical tourism. By ‘medical tourism’ we are referring to the practice whereby patients travel across international borders in order to privately access medical care [1]. The GHPMF included participants who were established medical tourism researchers from ten countries (Canada, China, Germany, Japan, Malaysia, The Netherlands, Singapore, South Korea, the United Kingdom and the United States) representing diverse academic disciplines (e.g., geography, business/marketing, political science, public policy, health systems management, and sociology), government (e.g., medical travel booster organisations) and industry (e.g., healthcare provider, healthcare marketing and branding, healthcare and health tourism market platform and association). It built on recent conferences organised in Madrid (2016), Wageningen and Leeds (2013) and Vancouver (2010) seeking to bring scholarly, policy and industry approaches to ‘medical tourism’ in conversation with one another [2, 3]. In this short report article, we identify key lessons from the forum that can inform the direction of future scholarly engagement with medical tourism. In so doing, we reference on-going scholarly debates about this global health services practice that have appeared in multiple venues.

GHPMF participants extensively discussed how ‘medical tourism’, a term widely popularised by the media, inadequately captures the diverse needs and experiences of people travelling outside of their countries of habitual residence to privately access medical treatment [4–6]. A more nuanced categorisation of the scope and scale of, and interactions between, patients travelling for treatment and the diverse array of stakeholders generating and responding to these patients’ needs and wants prior to, during and following their travels in their home, transit and destination countries is necessary. Such categorisation will enable global health researchers to transcend simple documenting of such diversity in order to begin to identify and effectively monitor basic variables that would permit, first, timely assessment of the economic and health equity impacts of travelling patients and, second, evidence-based discussion about and action regarding accountability for addressing these impacts. A key question for researchers is: who should be meaningfully involved in identifying and defining categories of those travelling across borders for health services and what risks exist if certain voices are underrepresented in such a process?

Governments around the world have been motivated to develop and promote their countries, cities and medical facilities as medical tourism destinations, seeking to tap into potential economic gains associated with the emerging sector (see, e.g., [7]). They routinely measure – and, with limited critique, both scholars and the media generally report – the number of medical travellers they receive per year as the key indicator of a medical tourism destination’s significance and its healthcare providers’ competence [8]. Yet, GHPMF attendees believe that such figures communicate very little and, because they are oftentimes arrived at through organization-specific counting processes that render them very difficult to compare, actually serve as hyperbolic obstacles to generating an objective overview of the scale of and diversity within the industry. Since, on the whole, travelling patients are thought to stay longer and spend more on average than conventional tourists [9], we argue that focus should instead be on calculating medical and non-medical spending and resource use associated with different types of medical treatment, and that this information be accessible for broader study of the industry. Researchers can work to produce new knowledge that can contribute to answering key questions such as: who does and does not ‘count’ as a medical tourist and what are the implications of such quantitative assessments?

Significant gaps in our knowledge about medical tourism exist. For example, while data is gathered in some countries on hospital revenue derived from treatment of foreign patients, scarce data is available on the indirect economic impacts of this practice (e.g., revenue for other medical and non-medical sectors, healthcare management innovation, employment generated, etc.), though its most significant economic benefits may well be for non-medical sectors [10, 11]. Likewise, in spite of significant concerns about the role of medical tourism in exacerbating health inequities in both Global South and Global North countries [12, 13], presentations at the GHPMF revealed that no research to date has evaluated or established a method with a coherent set of qualitative and quantitative measures to evaluate this exacerbation effect. Why have researchers not been able to address these pressing knowledge gaps? GHPMF attendees agree there is no singular answer. However, unlike the conventional tourism industry, there appears to be neither sufficient recognition of the utility among, nor adequate incentive for, public- and private-sector actors involved to align themselves to establish common definitions and data collection and reporting standards in order to identify economic and health equity impacts. Many GHPMF attendees agreed that this can be attributed to the fragmented nature of the medical tourism industry, characterised by a large pool of individual industry actors concerned with internal and international competition, and political sensitivities surrounding the distribution and management of finite healthcare resources.

Presentations at the GHPMF underscored how numerous governments have allocated significant funds to states, municipalities and medical service providers for the development of services and facilities to attract international patients, including offering land grants and fiscal incentives to build and renovate medical facilities, creating incentives to attract top medical expertise, importing cutting-edge medical equipment, promoting medical tourism and acquiring international accreditation for facilities. South Korea, for instance has spent more than USD 10 million per year on industry development since it identified medical tourism as an economic growth engine [14]. Yet the lack of widely-accepted definitions and their operationalisation into reliably and routinely measured variables effectively hinders bodies from measuring the return on their investments (ROI) and, in turn, from critically reviewing policy outcomes and more effectively executing or revising policy. GHPMF attendees agree this is problematic when we consider how medical tourism has been taken up by governments as an economic growth engine – with potential for generating and diversifying employment opportunities in struggling regional economies, boosting demand for locally-produced medical equipment and attracting biotechnology research and development – and been deployed in economy of scale arguments to justify the acquisition and geographic distribution of high-end medical technology that rarely would be required for use by local patients (e.g., proton-beam therapy). Examining economic efficacy thus calls political attention to the usefulness of policy facilitating medical tourism and to underlying motivations for pursuing such policy.

It is imperative that researchers more rigorously assess impacts not only at the national level but also at the supra-national level and the sub-national regional and municipal levels by asking nuanced questions such as: how do supra-national- and national-level polices and initiatives shape the ways in which medical tourism is unfolding in specific local centres and clinics? For example, certain cities and regions, like Penang in Malaysia or Seoul in South Korea, are disproportionately affected as destinations for travelling patients [15, 16]. Yet, no studies assessing their specific, local needs and concerns exist to date. Furthermore, simple comparison of national-level industry outcomes without considering the heavy investments made ultimately hinder our understanding of policy performance.

International agreements fostering trade liberalization, including the offshoring of medical-related services, have frequently been cited as key to advancing the globalisation of healthcare [17]. While such agreements have certainly further commoditised healthcare sectors around the world, the imaginary they conjure of a free market in a flat world has not (yet) come to be. Rather, presentations at the GHPMF underscored the continued significance of geographic and cultural-linguistic proximity in shaping transnational flows of travelling patients. Unless travelling for very specific or advanced procedures, long-haul travel is rare; people are more likely to seek care in neighbouring countries and places with which they already have established networks [18, 19]. A far more nuanced grasp of the needs, wants, material circumstances, origins and impacts of different travelling patients, therefore, would enable more targeted destination marketing efforts, doing away with wasteful promotion of entire countries as destinations for all types of medical treatments to largely undifferentiated imaginary pools of ‘medical tourists’. Likewise, a more nuanced grasp of where travelling patients are receiving care, the type of care, how they travel and are accommodated, and how they spend their time in destinations would enable far better destination and patient management. A useful question for researchers to explore is: what types of data can help us to articulate such nuanced perspectives and how can they be obtained and meaningfully incorporated into analyses? Meanwhile, the outcomes of such analyses can lead to more targeted development and allocation of healthcare and promotional resources and greater capacity to inform and engage local affected populations and supra-national regional bodies about the distribution and management of available healthcare resources and the benefits and challenges this poses.

## Conclusion

The GHPMF was held in Korea in October 2016. Forum attendees examined cutting-edge research findings regarding many facets of medical tourism and talked extensively about key research challenges that exist in this domain of scholarship. In this short report, we have characterised the scope of these examinations and discussions, and in doing so we have articulated specific questions that researchers must tackle in order to shape the policy dialogues and inform on-going debates about the potential for medical tourism to transform destination communities’ economies while benefitting or harming local people. We thus view this short report as an important time capsule that summarises the current state of medical tourism knowledge and policy as articulated by the thought leaders in attendance at the GHPMF while also pushing for research growth. We encourage the continued production of high-quality research in this field by scholars from a wide range of disciplines, as was observed at the GHPMF, and for continued dialogue between researchers about how we can advance the state of knowledge that informs contemporary thinking about this particular global health care mobility.

## Abbreviations

GHPMC: Global Healthcare Policy and Management Forum
ROI: Return on investment
